# Clinical Considerations

**DOI:** 10.1093/geroni/igaa057.3148

**Published:** 2020-12-16

**Authors:** George Kuchel

**Affiliations:** University of Connecticut, Farmington, Connecticut, United States

## Abstract

Varied physiological functions demonstrate increased heterogeneity with aging. Variability in force exertion and motor performance is higher in old age, with increased step-to-step gait variability indicating greater risk of falls and cognitive decline. Even in healthy older adults, renal function may show no change, slight decline, or marked decline. In contrast, heart rate variability declines with age, with decreased complexity and a higher risk of cardiac events. The risk of death, disease and disability varies among individuals with increasing heterogeneity with aging. As a result, frailty has been conceptualized as both as a phenotype and an accumulation deficit index, offering strong predictive validity when seeking to understand the heterogeneity of aging from the perspective of risk of mortality and physiologic dysregulation across different systems. Physical resilience defined as ability to maintain or restore function following exposure to stressors also demonstrates increased heterogeneity with aging.

